# Human Lactoferrin Synergizes with Etoposide to Inhibit Lung Adenocarcinoma Cell Growth While Attenuating Etoposide-Mediated Cytotoxicity of Human Endothelial Cells

**DOI:** 10.3390/biomedicines10102429

**Published:** 2022-09-28

**Authors:** Paulina Olszewska, Barbara Pazdrak, Marian L. Kruzel

**Affiliations:** 1 Department of Pharmaceutical Chemistry, Drug Analysis and Radiopharmacy, Faculty of Pharmacy, Medical University of Lodz, Muszynskiego 1, 90-151 Lodz, Poland; 2 Department of Melanoma Medical Oncology, The University of Texas MD Anderson Cancer Center, Houston, TX 77054, USA; 3 PharmaReview Corporation, Houston, TX 77054, USA

**Keywords:** recombinant human lactoferrin, etoposide, lung cancer, synergistic effect, combination strategy

## Abstract

Lung cancer continues to be the deadliest cancer worldwide. A new strategy of combining chemotherapeutics with naturally occurring anticancer compounds, such as lactoferrin, might improve the efficacy and toxicity of current chemotherapy. The aim of this study was to evaluate the effect of recombinant human lactoferrin (rhLf) in combination with etoposide on anticancer activity in human lung adenocarcinoma cells. In addition, we examined the impact of rhLf on etoposide-induced cytotoxicity of human endothelial cells. We found that treatment of A549 cells with a combination of etoposide and rhLf resulted in significantly greater inhibition of cancer cell growth as compared to etoposide alone. The combination repressed cancer cell growth by cell cycle arrest in the G2/M phase and induction of apoptosis. In contrast to cancer cells, rhLf did not affect endothelial cell viability. Importantly, rhLf significantly diminished the etoposide-induced cytotoxicity of endothelial cells. Analysis of the type of drug interaction based on combination index value showed that rhLf synergized with etoposide to induce anticancer activity. The calculated dose reduction index indicated that the combination treatment reduced a 10-fold of etoposide dose to achieve the same anticancer effect. Our data demonstrate that rhLf enhanced the anticancer activity of etoposide and diminished etoposide-induced cytotoxic effect in endothelial cells.

## 1. Introduction

Cancer is still one of the greatest public health challenges worldwide. Despite the recent advances in targeted and immune therapies, the survival rate of lung cancer patients is still very low (18.6%). Furthermore, lung cancer is not only the leading cause of cancer-related deaths with about 1.8 million mortalities in 2020 but is also the most frequently diagnosed malignancy in the world [1,2]. Approximately 85% of lung cancer patients have been diagnosed with non-small cell lung cancer (NSCLC). Among NSCLC cases, lung adenocarcinoma is the most prevalent and aggressive histological subtype, accounting for approximately 40% [3]. Clinical studies have shown that only a subset of patients responds to checkpoint inhibitors or targeted therapies and patients often develop the acquired resistance after an initial response [4,5,6]. Therefore, traditional chemotherapy remains the option for the majority of lung cancer patients despite its toxicity [7]. One of the most commonly used regimens in standard chemotherapy for patients with inoperable NSCLC is etoposide [8,9,10,11]. Etoposide, a topoisomerase II inhibitor, was initially the most common therapeutic option for lung cancer, either singly or in combination with doxorubicin [12] or cisplatin [13]. However, inevitable side effects restricted the effectiveness of chemotherapy.

Thus, the development of non-toxic natural products with anticancer activity has been one of the most intense research interests, owing to the reduced risks of chemoresistance and the absence of side effects [14].

Lactoferrin (Lf) is a natural iron-binding glycoprotein, a member of the transferrin family that was first identified in bovine milk. Many studies on the biological activities of Lf have shown its multiple beneficial effects, including antiviral, antibacterial, antiparasitic, and antioxidant properties [15,16]. Lf is also involved in the regulation of immune responses and redox homeostasis [17,18,19,20,21]. Importantly, many studies confirmed the anticancer activity of Lf against human cancers in a variety of in vitro and in vivo models without toxicity [22,23,24,25,26,27,28]. Notably, the majority of the research on anticancer effects of Lf was performed using bovine Lf (bLf) [14,23,29,30,31] which exhibits a different glycosylation profile and shares about 70% sequence homology with human Lf [29,32].

Glycosylation is a highly regulated post-translational modification process that allows the covalent addition of glycan moieties to the specific amino acids of a protein. Glycosylation directly impacts protein structure and modulates its biological functions [33,34,35]. For example, the oligosaccharide component of glycoprotein plays a role in cell adhesion and receptor activation. Moreover, the glycan profile is critical for the determination of protein immunogenicity and antigenicity [33,34,36]. Thus, bLf cannot be used for parenteral therapy. To explore the beneficial effects of human Lf, a few forms of recombinant human lactoferrin (rhLf) were obtained from different expression systems exhibiting a variable glycosylation profile [37]. Orally administrated Talactoferrin, a form of rhLf expressed in the fungus *Aspergillus*, was evaluated in clinical trials in patients with refractory NSCLC [36,38]. However, rhLfs produced in fungal and yeast expression systems exhibit high levels of mannose N-linked glycans as compared to the natural human Lf. This different glycosylation profile of Talactoferrin may induce immunogenic responses, and therefore cannot be used for parenteral clinical application [39,40].

Notably, in the current study, we used rhLf expressed in the mammalian system, which produces rhLf with a glycosylation pattern compatible with the natural Lf isolated from human milk [40]. Recently, we have demonstrated that this rhLf possesses selective anticancer activity against human lung adenocarcinoma with no cytotoxic effect on normal human bronchial epithelial cells [28]. Thus, this rhLf might be a prototype for the development of a non-toxic natural anticancer agent suitable for an intravenous application, as well as for intratumoral injections.

It is well recognized that many anticancer drugs are most effective when used in combination in order to overcome off-target toxicity and resistance to treatment. Therefore, in this study, we aimed to evaluate the potential benefits of a new combination strategy of etoposide with rhLf on inhibition of human lung adenocarcinoma cell growth compared with etoposide alone. In addition, we investigated whether rhLf affects etoposide-induced cytotoxicity in human endothelial cells. Our results showed that rhLf synergized with etoposide to inhibit human lung adenocarcinoma cell growth by cell cycle arrest in the G2/M phase and induction of apoptosis, while diminished etoposide-mediated cytotoxicity of endothelial cells. Importantly, the quantitative analysis of the type of drug interaction showed that combining rhLf with etoposide reduced a 10-fold etoposide dose to achieve the same anticancer effect. These results provide evidence for a potential clinical benefit of the combination of rhLf with chemotherapy.

## 2. Materials and Methods

### 2.1. The Human Lactoferrin

Recombinant human lactoferrin (rhLf) was supplied by PharmaReview Corporation (Houston, TX, USA) as the lyophilized powder (95% purity by SDS PAGE; <15% iron-saturated; <0.5 endotoxin units mg^−1^). The rhLf was expressed in CHO cells, followed by the scale-up of production and purification as described by Kruzel et al. [40].

### 2.2. Cell Culture

The human lung adenocarcinoma cell line (A549) was purchased from the European Collection of Cell Cultures (ECACC). The A549 cells were cultured in DMEM (Biowest, Nuaille, France) containing 10% heat-inactivated fetal bovine serum, FBS (Biowest, Nuaille, France), and 100 units/mL penicillin and 100 μg/mL streptomycin (Biowest, Nuaille, France). Human umbilical vein endothelial cells (HUVEC) were obtained from Lonza and cultured in EGM-2 medium (Lonza, Basel, Switzerland) supplemented with FBS and growth factors (Bullet Kit, Lonza, Bael, Switzerland). Both cells were cultured in standard cell conditions (37 °C, 5% carbon dioxide, and 90% humidity) in an incubator (HeraCell iVios160, ThermoFisher Scientific, Waltham, MA, USA).

### 2.3. Preparation of Compounds Solution for In Vitro Biological Assays

An amount of 0.1 M stock solution of etoposide (Sigma-Aldrich, Saint Louis, MO, USA) was prepared in DMSO, aliquoted, and stored at −20 °C. Etoposide solution was freshly diluted to an appropriate concentration in a culture medium prior to being used in the in vitro assays. rhLf (PharmaReview Corporation, Houston, TX, USA) solution (stock 1 mg/mL) was prepared in a culture medium before the experiments.

Control cells were treated with DMSO alone in the highest corresponding concentration of etoposide.

### 2.4. Cell Viability Assay

Cell viability was assessed using a colorimetric WST-1 assay (Takara, Kusatsu, Japan) according to the manufacturer’s instructions. The amount of formazan dye formed by mitochondrial dehydrogenase present in viable cells directly correlates with the number of living cells. A549 cells were seeded in a 96-well plate at a density of 5000 cells per well and cultured for 24 h. Next, the cells were treated with various concentrations of rhLf (from 0.2 to 1 mg/mL), etoposide (10 and 20 µM), or a combination of rhLf and etoposide. After 24 h of treatment, 10 µL of WST-1 solution was added to each well and the absorption of formazan was measured after 1 h and 2 h incubation in A549 cells and HUVEC cells, respectively, using a microplate reader (iMARK, Bio-Rad, Hercules, CA, USA) at 450 nm. Cellular viability was expressed as a percentage of the control cells, which constituted 100% viability.

### 2.5. Cell Density and Morphology

The effects of rhLf, etoposide, or a combination on cell density and the morphology of A549 and HUVEC cells were examined using an inverted microscope with phase contrast (Opta-Tech, software OptaView 7, Warsaw, Poland). A549 cells or HUVEC cells were photographed after the indicated time of treatment at 100× magnification.

### 2.6. Analysis of the Type of Interaction between Lactoferrin and Etoposide

ComboSyn software (Combosyn Inc., Paramus, NJ, USA, www.combosyn.com, accessed on 29 November 2021), based on the Chou-Talalay method [41,42,43], was employed to determine the type of interaction between the rhLf and etoposide in A549 and HUVEC cells using WST assay results. This method utilizes the median-effect equation derived from the enzyme kinetics model, which provides the theoretical basis for the combination index (CI)-isobologram equation [42]. CI-isobologram equation allows quantitative determination of the type of drug interaction based on CI values, where CI  <  1 indicates synergism; CI in the range 0.9–1.1 shows additivity; CI  >  1 indicates antagonism. The calculated CI values and the fraction affected (Fa) for each dose of tested drugs were used to generate the Fa-CI plots. Dose reduction index (DRI) was also analyzed to generate a Fa-DRI plot. DRI denotes folds of dose reduction that are allowed for each drug due to synergism when compared with the dose of each drug alone. DRI > 1 indicates favorable dose reduction, while DRI = 1 shows no dose reduction, and DRI < 1 represents unfavorable dose reduction [42,43,44].

### 2.7. Cell Cycle Analysis

A549 cells were seeded in six-well plates at a density of 200,000 cells per well and cultured for 24 h. Cells then were treated with rhLf, etoposide, or the combination for 24 h. Next, the collected cells were washed with cold DPBS (Biowest, Nuaille, France) and fixed in 70% ethanol at 4 °C overnight. After washing in DPBS, the cells were incubated with ribonuclease (Sigma-Aldrich, Saint Louis, MO, USA) at a final concentration of 100 µg/mL at 37 °C for 30 min, followed by the cell staining with propidium iodide (PI) (Sigma-Aldrich, Saint Louis, MO, USA) at the final concentration of 50 µg/mL. The stained cancer cells were analyzed by flow cytometry using CytoFlex cytometer (Beckman Coulter, Brea, CA, USA). Cell cycle distributions were assessed in 20,000 cells collected from each sample to calculate the percentage of cells in each cell cycle phase. The results were presented as the mean ± SD of the three independent experiments.

### 2.8. Annexin V/PI Assay/Apoptosis Analysis

To identify and quantify apoptotic cells in response to different treatments we performed double staining of cells with Annexin V and PI using the AnnexinV/PI apoptosis detection kit (Biolegend, San Diego, CA, USA), according to the manufacturer’s instructions.

A549 cells were seeded in 6-well plates at a density of 300,000 cells per well. After 24 h culture, the cells were treated with rhLf, etoposide, or the combination for 24 h. After cell washing with Cell Staining Buffer (Biolegend, San Diego, CA, USA), the cells were resuspended in Annexin Binding Buffer and stained with Annexin V and PI for 15 min at room temperature in the dark. The stained cells were analyzed by flow cytometry using CytoFlex cytometer (Beckman Coulter, Brea, CA, USA). Cells were gated according to PI and Annexin V staining, and the results were quantified using Kaluza 2.1 software (Beckman Coulter, Brea, CA, USA). Cells positive for Annexin V only represented early apoptotic, cells positive for PI only were considered necrotic and cells double positive indicated late apoptotic cells. The data were presented as the mean ± SD of three independent experiments.

### 2.9. Statistical Analyses

Data were obtained from at least three separate experiments and all results were expressed as mean ± SD. Statistical analysis of the results was completed using Statistica 13.3 software (StatSoft). The normal distribution of continuous variables was verified with the Shapiro–Wilk test. The paired t-test was used for statistical comparisons of all variables with a normal distribution. The variables with non-normal distributions were compared using the Wilcoxon signed-rank test. *p* less than 0.05 was considered statistically significant (* *p* < 0.05).

## 3. Results

### 3.1. Effect of rhLf in Combination with Etoposide on Lung Cancer Cell Growth

**Figure 1:**
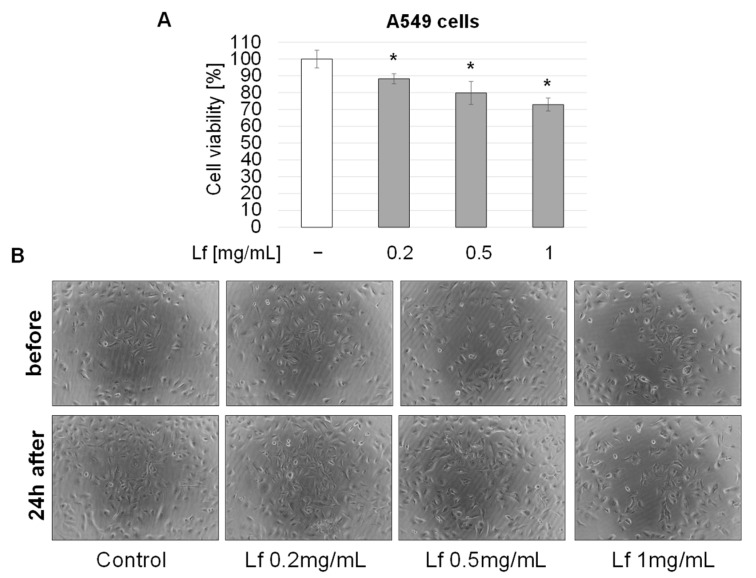
Effect of recombinant human lactoferrin (rhLf) on lung cancer cell growth. (**A**) A549 cells were cultured in the presence of rhLf (Lf) at indicated concentrations for 24 h. Viable cells were quantified by a WST assay. The results were expressed as a percentage of control cells and presented as the mean ± SD of three experiments performed in duplicate; (**B**) representative cell images before and 24 h after treatment visualized the effect of rhLf on cancer cell growth. (100× magnification). * *p* < 0.05 compared to the control.

A549 cells were treated with three different concentrations of rhLf for 24 h and cell viability was determined by WST assay. The amount of formazan generated by the activity of mitochondrial dehydrogenase is directly proportional to the number of alive cells. Figure 1A showed that rhLf displayed dose-dependent inhibition of cancer cell growth. For example, the exposure of A549 cells to 1 mg/mL of rhLf significantly decreased cell growth by 27.08 ± 3.89% as compared to the control (Figure 1A). The cell images before and 24 h after treatment visualized the lower density of cells cultured in the presence of rhLf compared to the density in the control cells after 24 h (Figure 1B).

**Figure 2:**
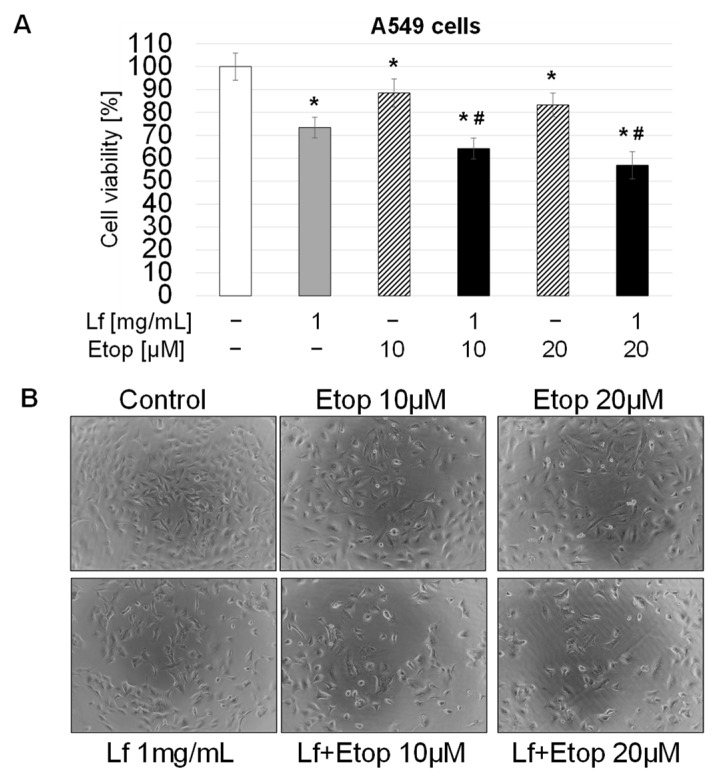
Effect of rhLf in combination with etoposide on A549 cell growth. (**A**) A549 cells were cultured in the presence of rhLf (Lf) and etoposide (Etop) alone or in the combination of both at indicated concentrations for 24 h and viable cells were quantified by a WST assay. The results were calculated as a percentage of control cells and expressed as the mean ± SD of three experiments performed in duplicate. (**B**) Visualization of the effect of rhLf, etoposide alone or the combination on cancer cell growth and cell morphology. Representative phase-contrast cell images are shown after 24 h treatment (100× magnification). * *p* < 0.05 compared to the control. ^#^ *p* < 0.05 compared to etoposide alone at the same concentration.

To evaluate whether rhLf enhances the etoposide-induced anticancer effect, A549 cells were treated with a combination of rhLf and etoposide or a single agent for 24 h. For the combination treatment, we selected 1 mg/mL of rhLf that exhibited the greatest effect, and we used etoposide at low concentrations of 10 μM and 20 μM that induced a modest effect on inhibition of cancer growth (11.5% and 16.7%, respectively) (Figure 2A). We found that exposure of A549 cells to a combination of rhLf and etoposide significantly enhanced inhibition of cancer cell growth as compared to etoposide alone at both tested concentrations (Figure 2A). For example, treatment of cells with rhLf in combination with 10 μM of etoposide suppressed cell growth by 35.8 ± 4.6%, whereas treatment of cells with the same concentration of etoposide alone inhibited cell growth only by 11.5 ± 6.1% (Figure 2A). The cell images also visualized the lower density of A549 cells treated with the combination rhLf and etoposide as compared to etoposide alone for each concentration (Figure 2B). These results demonstrated that rhLf significantly increased the anticancer effect of etoposide in human lung adenocarcinoma cells.

### 3.2. Synergistic Anticancer Effect of Etoposide in Combination with rhLf

**Figure 3:**
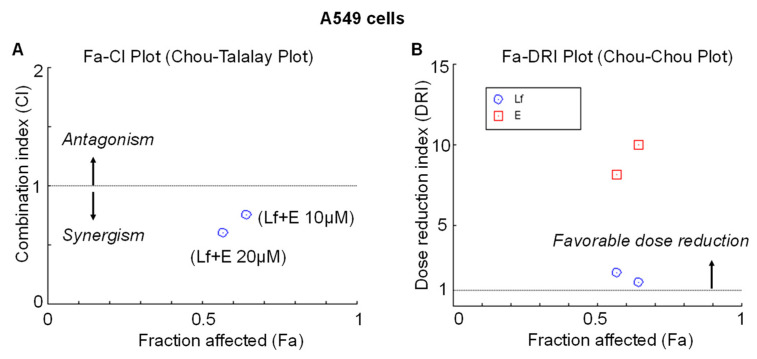
Analysis of the type of interaction between rhLf and etoposide in A549 cells by the CompuSyn software. (**A**) Fa-CI plot (Chou-Talalay Plot) showing a synergistic effect of the combination rhLf (Lf) and etoposide (E) for each fraction affected. CI  <  1 indicates synergism; CI  =  1 indicates additivity; CI  >  1 indicates antagonism; (**B**) Fa-DRI Plot (Chou-Chou Plot) represents an index of dose reduction for each fraction affected. DRI  >  1 indicates favorable dose reduction; DRI  <  1 indicates unfavorable dose reduction.

To determine the type of interaction between rhLf and etoposide in lung cancer cells, we used the CompuSyn software (The ComboSyn, Inc., 2005) to calculate the combination index (CI) values of each dose. CompuSyn software defines synergy as a CI value less than 1 and values more than one specifies antagonism. As shown by the Fa-CI plot, the calculated CI values for the combination of rhLf (1 mg/mL) with etoposide at 10 µM and 20 µM were 0.758 and 0.604, respectively, for corresponding the fraction inhibition of Fa = 0.64 and Fa = 0.57, indicating the synergistic effect (Figure 3A). In addition, we estimated the dose reduction index (DRI) that measures a fold reduction of the dose for each drug in a synergistic combination at a given effect level when compared with the dose of each drug alone. A DRI value of more than 1 indicates a favorable dose reduction. The estimated DRI values for etoposide at 10 µM and 20 µM were 10.0 and 8.2, respectively, indicating folds of the dose reduction of etoposide in a synergistic combination with rhLf to induce inhibition of cancer cell growth by 36% (Fa = 0.64) and 43% (Fa = 0.57), respectively (Figure 3B). The other words, the concentration of 100 µM and 160 µM etoposide alone would be required to achieve the same anticancer effect (36% and 43%), thus the combination treatment reduced a 10- and 8-fold dose of etoposide, respectively. Notably, once the dose of etoposide is reduced, the toxicity of the drug will ultimately decrease. Together, quantitative determination of the drug interaction indicated that the combination of rhLf and etoposide displayed a synergistic anticancer effect against human lung adenocarcinoma cells at the tested concentrations.

### 3.3. Effect of rhLf in Combination with Etoposide on Cancer Cell Cycle Progression

**Figure 4:**
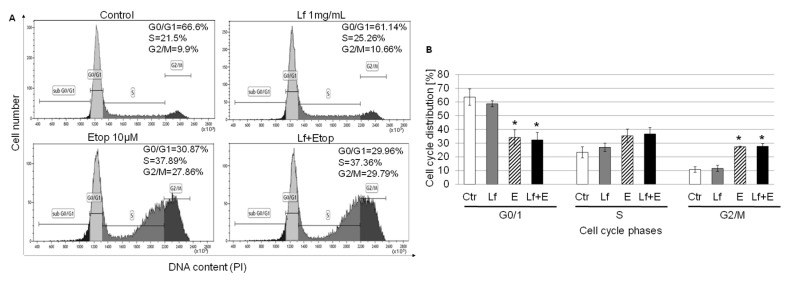
Effect of rhLf in combination with etoposide on A549 cell cycle progression. A549 cells were treated with rhLf (Lf) and etoposide (E) alone or the combination (Lf + E) at indicated concentrations for 24 h. (**A**) Representative histograms show the cell cycle distribution at G0/1, S, and G2/M phases in the control and treated cells. (**B**) The bar graph indicates the percentage of cells at each phase of cell cycle in the control cells (Ctr) and cells after indicated treatments. The data are expressed as the mean ± SD of three experiments. * *p* < 0.05 compared to the control.

To determine the mechanisms responsible for lactoferrin and etoposide-induced inhibition of cancer cell growth, we examined the effect of the combination of rhLf and etoposide on A549 cell cycle distribution. Flow cytometry analysis based on the fluorescence intensity of PI-stained cancer cells displayed separation of cells in the G0/1, S, and G2/M phases. Figure 4A shows representative profiles of cell cycle distribution in the control cells and cells exposed to rhLf and etoposide alone or the combination for 24 h.

The results showed that treatment of A549 cells with rhLf at 1 mg/mL concentration had no significant effect on cell cycle progression. We observed only a slight reduction in cell number in the G1 phase (about 5%). In contrast, etoposide treatment induced a significant decrease in the percentage of cells in the G1 phase and an accumulation of cells in the G2/M phase of the cell cycle. Compared with the control, the cell population in the G2/M phase increased from 10.80 ± 2.02% to 27.42 ± 0.40%, with a comparative decrease in the percentage of cells in the G1 phase from 63.57 ± 5.98% to 34.20 ± 5.60 in response to etoposide (Figure 4B). The combination of rhLf and etoposide treatment also induced growth arrest at the G2/M phase of the cell cycle, with a corresponding reduction of cells in the G1 phase compared to the effect of etoposide alone (Figure 4A,B).

### 3.4. Effect of rhLf in Combination with Etoposide on Cancer Cell Apoptosis

**Figure 5:**
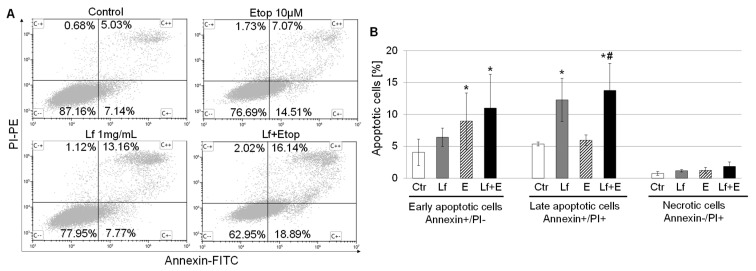
Effect of rhLf in combination with etoposide on A549 cell apoptosis. (**A**) Representative plots of flow cytometry analysis of Annexin V and PI staining of the control and cells after treatment with rhLf (Lf) and etoposide (E) alone or the combination (Lf + E) at indicated concentrations for 24 h. Viable cells (Annexin−/PI−), early apoptotic cells (Annexin+/PI−), late apoptotic cells (Annexin+/PI+), necrotic cells (Annexin−/PI+). (**B**) The bar graph shows the quantification of early and late apoptotic cells, and necrotic cells in response to indicated treatments. The values represent the mean ± SD of four experiments. * *p* < 0.05 compared to control (Ctr) and ^#^ *p* < 0.05 compared to etoposide alone (E).

The initiation of the apoptosis process may be induced by cell cycle arrest. Therefore, we quantified apoptotic cells by Annexin V and PI staining to evaluate whether the repression of lung cancer cell growth by the combination treatment is associated with an induction of apoptosis. Annexin V binds to phosphatidylserine on the external leaflet of the plasma membrane during early apoptosis, thus early apoptotic cells will exclude PI due to preserving the plasma membrane integrity. In contrast, late apoptotic cells and necrotic cells will be PI-positive. The representative cytograms of flow cytometry analysis from the control and treated cells with rhLf, etoposide, and the combination visualized changes in the percentage of living (Annexin−/PI−), early apoptotic (Annexin+/PI−), late apoptotic (Annexin+/PI+) and necrotic cells (Annexin−/PI+) in response to each treatment (Figure 5A). The results illustrated that 24 h treatment of A549 cells with rhLf increased about two-fold the percentage of late apoptotic cells as compared to the control (12.27 ± 3.39% vs. 5.36 ± 0.31%, respectively) (Figure 5B). In contrast, at the same time etoposide induced a two-fold increase in the percentage of early apoptotic cells as compared to the control (8.95 ± 4.41% vs. 4.05 ± 2.06%) but had no effect on the number of late apoptotic cells (Figure 5B). Interestingly, treatment of cancer cells with the combination resulted in a significant increase in the percentage of both early (2.5-fold) and late (2.5-fold) apoptotic cells in comparison with the control or etoposide alone for late apoptotic cells. Overall, data demonstrated that rhLf enhanced etoposide-induced apoptosis in human lung adenocarcinoma cells.

### 3.5. Antagonistic Effect of rhLf on Etoposide-Induced Cytotoxicity of Human Endothelial Cells

In general, the efficacy of chemotherapy in patients is restricted by its inevitable side effects. Therefore, the second aim of the current study was to investigate whether rhLf attenuates the cytotoxic effect of etoposide on normal human cells. To assess etoposide-induced cytotoxicity, we selected HUVEC cells, which represent human endothelial cells.

**Figure 6:**
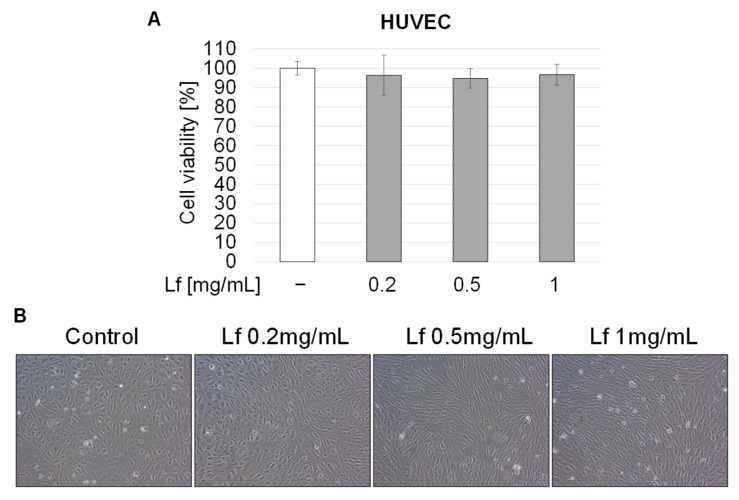
Effect of rhLf on viability of human endothelial cells. (**A**) Confluent HUVEC cells were cultured in the presence of indicated concentrations of rhLf (Lf). Cell viability was quantified by a WST assay after 72 h. The data are expressed as the mean ± SD of three experiments performed in duplicate. (**B**) Visualization of the effect of rhLf on confluent and morphology of HUVEC cells. Representative phase-contrast cell images are shown after 72 h treatment (100× magnification).

**Figure 7:**
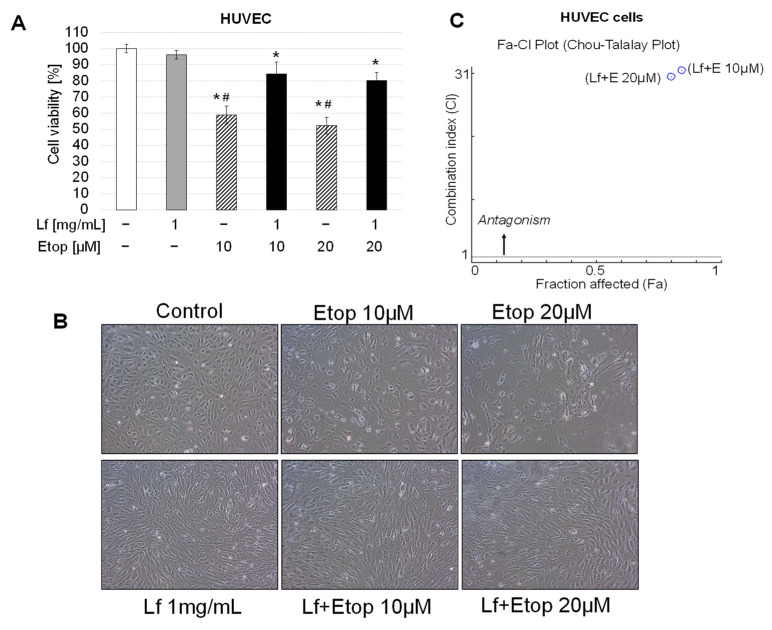
Effect of rhLf on etoposide-induced cytotoxicity of human endothelial cells. (**A**) Confluent HUVEC cells were cultured in the presence of rhLf (Lf) and etoposide (Etop) alone or the combination at indicated concentrations. Cell viability was quantified by a WST-1 assay after 72 h. The data are expressed as the mean ± SD of three experiments performed in duplicate. (**B**) Visualization of the effect of etoposide alone and in combination with rhLf on confluent, cell morphology, and density of HUVEC cells. Representative phase-contrast cell images are shown after 72 h treatment (100× magnification). (**C**) Analysis of the type of interaction between rhLf and etoposide in HUVEC cells by the CompuSyn software. Fa-CI plot (Chou-Talalay Plot) showing the antagonistic effect of combination rhLf and etoposide (Lf + E) for each fraction affected. CI  <  1 indicates synergism; CI  =  1 indicates additivity; CI  >  1 indicates antagonism. * *p* < 0.05 compared to the control and ^#^ *p* < 0.05 compared to the combination treatment at the same concentration of etoposide.

Initially, we examined the effect of rhLf on the viability of endothelial cells. The confluent HUVEC cells were cultured in the presence of rhLf at the same tested concentrations for 72 h. The results showed that rhLf up to 1 mg/mL did not affect significantly endothelial cell viability (Figure 6A). Furthermore, when cells were cultured in the presence of rhLf, the cell images visualized healthy endothelial cell confluent with tight cell-cell junctions comparable to the control cells (Figure 6B). We observed that rhLf at 0.5 mg/mL and 1 mg/mL concentrations induced slight elongation of endothelial cells (Figure 6B). In contrast, exposure of HUVEC cells to etoposide at 10 μM and 20 μM concentrations significantly decreased cell viability by 41.12 ± 5.51% and 47.73 ± 5.13%, respectively (Figure 7A). In contrast to rhLf-treated cells, the images of endothelial cells cultured with etoposide for 72 h visualized profound changes in cell morphology characterized by loss of endothelial cell confluency, absence of tight cell-cell junctions, and an overall decrease in the number of attached cells (Figure 6B).

Next, we evaluated the effect of rhLf in combination with etoposide on endothelial cell viability at the same concentrations that were used for the treatment of cancer cells. Importantly, we found that treatment of HUVEC cells with the combination resulted in a significant increase in endothelial cell viability as compared with etoposide alone at both tested concentrations (Figure 7A). For example, the combination treatment enhanced the cell viability from 58.9 ± 5.5% to 84.3 ± 7.3% and from 52.3 ± 5.1% to 80.2 ± 5.0% in comparison with etoposide alone at 10 μM and 20 μM, respectively. Notably, the images of cells after treatment with the combination showed the confluent of endothelial cells with cell-cell junctions comparable to the control and rhLf-treated cells (Figure 7B). Furthermore, we determined the type of interaction between rhLf and etoposide in endothelial cells based on the analysis of CI values of each dose using the CompuSyn software. CI value less than 1 defines synergy and CI value more than 1 defines antagonism. The calculated CI values for the combination of rhLf (1 mg/mL) with etoposide at 10 µM and 20 µM were 30.5 and 29.5, respectively, indicating a strong antagonistic effect (Figure 7C). The results clearly demonstrated that rhLf significantly diminished the etoposide-induced cytotoxicity of human endothelial cells.

## 4. Discussion

Overall, the main limitation of standard chemotherapy is profound side effects resulting in drug-induced cytotoxicity on normal cells. In this study, we aimed to determine the potential benefits of a new combination strategy of a chemotherapeutic, etoposide with rhLf, a natural immunomodulator having anticancer properties. In this study, we demonstrated that rhLf synergized with etoposide to enhance its anticancer effects in lung adenocarcinoma cells and attenuated etoposide-mediated cytotoxicity of human endothelial cells. These findings are clinically significant because they indicate the therapeutic potential of this new combination strategy for improving the efficacy and toxicity profile of chemotherapy.

Malignancy is growing into a major public health problem worldwide. It has been estimated 19.3 million new cancer cases and almost 10 million cancer-related mortalities in 2020 [1,2]. The introduction of checkpoint inhibitors and targeted therapy represents major advances in cancer treatments. However, clinical studies showed the majority of patients do not respond to these therapies or develop resistance after an initial tumor regression [4,5,6]. Although they are generally well tolerated, they are not without toxicities [45,46,47]. Lung cancer is the leading cause of cancer death. In particular, lung adenocarcinoma is the most aggressive form of NSCLC with a rapid increase in prevalence [48,49].

Unfortunately, standard chemotherapy is still a key modality in today’s practice of clinical oncology for patients with advanced malignancies [50]. Etoposide is a potent chemotherapeutic agent used in the management and treatment of various cancers, including lung, testicular, prostate, bladder, and stomach cancer [12,13]. The mode of action of this agent relies on the inhibition of DNA synthesis as a result of a complex formation with topoisomerase II and DNA. This complex induces breaks in double-stranded DNA and subsequently prevents DNA repair. Accumulation of DNA damage inhibits entry into the mitotic phase of the cell cycle resulting in the induction of the apoptosis process [51]. It is well recognized that the efficacy of chemotherapeutic agents depends on the dose and time of exposure. Therefore, the dose-related side effects limit the efficacy of chemotherapy in treated patients.

Lf is a natural glycoprotein with numerous biological roles, including modulation of immune responses, and has anticancer activities [22,24]. Recently, we have shown that a novel form of rhLf that exhibits a glycosylation profile compatible with the natural human Lf, has selective anticancer effects against human lung adenocarcinoma without cytotoxicity on normal human bronchial epithelial cells [28]. Similarly, bLf and rhLf derived from yeast showed anticancer activity in squamous cell carcinoma and breast cancer, respectively, without cytotoxic effects on the equivalent of normal human cells [31,52]. This is a very important aspect of Lf since the selective cytotoxicity against cancers is the most important feature of any antitumor drug. A few potential mechanisms responsible for the selective anticancer effect of Lf have been proposed. Some studies demonstrated the expression of Lf receptors on the surface of tumor cells. For example, most cancer cells express high levels of glycosaminoglycans and proteoglycans, as well as sialic acids, which have the capability of interacting with Lfs [53,54]. Lf is a highly glycosylated protein and the natural human Lf contains three potential *N*-glycosylation sites. It has 3 mono-, 1 bi-, and 1 tri-fucosylated *N*-glycans, and a lot of sialylated oligosaccharides, and no significant amounts of high-mannose structures [55]. It is well recognized that the oligosaccharide component of glycoprotein plays a significant role in biological functions, including activation of receptors [33,34,35]. Thus, a primary interaction of Lf with specific receptors on the cancer cell surface may be responsible for the direct recognition of cancer cells and selection between normal cells. Consequently, anticancer specificity and selectivity of Lfs could be mediated by binding to these receptors and activation of signaling pathways leading to cancer cell death [56]. Moreover, cancer cells overexpress the genes implicated in iron uptake and demand higher concentrations of iron to grow [57]. Therefore, Lf is able to selectively inhibit cancer cell growth by scavenging free iron and subsequently decreasing its availability. Additionally, Lf could be implicated in an iron-related mechanism of cancer cell death via ferroptosis [58,59]. Furthermore, cancer cells overexpress plasma membrane proton pump V-ATPase, and inhibition of its activity leads to a reduction in the acidity in the tumor microenvironment. Interestingly, bLf specifically inhibits the action of this proton pump only in highly metastatic cancer cells [60]. Clinical studies on orally administered Talactoferrin, rhLf derived from a fungus, showed anticancer activity against NSCLC tumors without drug-related side effects [36,38].

Considering the limited effectiveness of current therapeutic protocols for lung cancer, the development of novel biotherapeutics with the ability to specifically target cancer cells, with little or no cytotoxicity on normal cells is highly desirable. Therefore, rhLf is a potent candidate for the development of new therapeutic strategies for lung cancer treatment. Because rhLf is a natural and non-toxic agent, it could be delivered intravenously or directly into the tumor with no risk of an immune adverse reaction.

The main aim in the development of drug combination therapy is to achieve a synergistic therapeutic effect to reduce the dose of the drug, and minimize toxicity or delay the induction of drug resistance [42]. In the current study, we explored the potential benefits of rhLf in a combination with etoposide on anticancer activity and evaluated the impact of rhLf on etoposide-induced cytotoxicity in human endothelial cells in comparison to the effect of etoposide alone. We found that the combination treatment displayed a greater effect on inhibition of adenocarcinoma cell growth than etoposide alone. Overall, the combination of rhLf and etoposide suppressed cancer cell growth by cell cycle arrest at the G2/M phase and the induction of apoptosis. This effect is consistent with well described the mode of action of etoposide, which induces DNA damage and prevents entry into the mitotic phase of cell division leading to the initiation of apoptosis. Etoposide acts primarily in the G2 and S phases of the cell cycle [51]. Our results showed that inhibition of cells proliferation in the S and G2/M phases of cell cycle with the combination treatment was comparable to the effect of etoposide alone. However, treatment of lung cancer cells with the combination significantly increased the percentage of early apoptotic cells as compared to rhLf alone, and the same time enhanced the frequency of late apoptotic cells as compared to etoposide alone. Thus, analysis of apoptotic cells by Annexin V/PI staining showed that etoposide in the combination with rhLf had a greater effect on stimulation of cancer cell death compared to either agent alone. Moreover, these results suggested that etoposide and rhLf induced cancer cell death by different molecular mechanisms. It may also explain why the combination of rhLf and etoposide potentiated this anticancer effect.

To define the type of drug interaction between rhLf and etoposide in lung cancer cells, we calculated the combination index (CI) values of each dose. This quantitative determination indicated that the combination of rhLf and etoposide displayed a synergistic anticancer effect. One major aim for achieving synergy in drug combination is that it allows dose reduction for the therapeutic effect. The beneficial consequence of dose reduction of the drug is the attenuation of drug toxicity toward the host. The estimated dose reduction index (DRI) values for etoposide at 10 µM and 20 µM were 10.0 and 8.2, respectively, indicating folds of the dose reduction of etoposide in a synergistic combination with rhLf. It means that concentrations of 100 µM and 160 µM etoposide alone would be required to achieve the same anticancer effect. Thus, rhLf in the combination treatment reduced 10- and 8-fold the dose of etoposide.

Endothelial cells form the barrier between blood vessels and tissue and control the flow of different substances and fluids. Moreover, they play an important role in many physiological functions, including coagulation, regulation of vessel permeability and vascular tone, or recruitment of leukocytes [61,62]. Thus, drug-related endothelium damage during chemotherapy may induce profound side effects on the whole body. For this reason, in the present study we also evaluated whether rhLf protects human endothelial cells from etoposide-mediated cytotoxicity. Consistent with the effect of this rhLf on normal human bronchial epithelial cells [28], exposure of endothelial cells to rhLf at the concentration that induces anticancer activity did not affect its viability. As expected, etoposide induced profound endothelial cell cytotoxicity with complete disruption of cell–cell junctions and decrease cell viability in dose-dependent manner. We showed for the first time that rhLf significantly diminished the etoposide-mediated cytotoxic effect in human endothelial cells. We also determined the type of drug interaction between rhLf and etoposide in endothelial cells based on the analysis of CI values of each dose. The calculated CI values for the combination of rhLf with etoposide indicate a strong antagonistic effect. The results demonstrated that rhLf significantly diminished the etoposide-induced cytotoxicity of normal human endothelial cells.

## 5. Conclusions

The development of more effective therapeutics or strategies that discriminate between normal and cancer cells to minimize adverse events is one of the major challenges in today’s oncology. The novel rhLf that resembles the glycan structure of the natural human Lf has the ability to selectively target the cancer cells. Our results showed that rhLf synergized with etoposide to inhibit human lung adenocarcinoma cell growth and reduced several folds of the etoposide dose to achieve the same anticancer effect. Importantly, rhLf significantly diminished the cytotoxicity of human endothelial cells induced by etoposide. Our study provides evidence that a new strategy of combining rhLf with chemotherapeutic could have clinical benefits by improving efficacy and reducing toxicity.

## Data Availability

Data are contained within the article.
